# Finding Well-Being in the Stands: High-Involvement Fandom and Subjective Well-Being Among Female Spectators in Korea’s Record-Breaking KBO League

**DOI:** 10.3390/bs16091559

**Published:** 2026-09-02

**Authors:** Jaeyoon Kwon, Sangback Nam, Kyunghan Yoon

**Affiliations:** Department of Sports Science, Hanyang University, ERICA, Ansan 15588, Republic of Korea; heykwon@hanyang.ac.kr

**Keywords:** female fandom, secondary qualitative analysis, KBO League, team identification, subjective well-being

## Abstract

This study examines the psychological and social processes through which high-involvement sports fandom is associated with the subjective well-being of female spectators in South Korea’s professional KBO baseball league. Utilizing a secondary qualitative analysis informed by grounded-theory coding procedures, we investigate the dynamic processes, interaction strategies, and sociopsychological factors through which female spectators experience high-involvement fandom and how this engagement relates to their well-being. Data originally collected through in-depth semi-structured interviews with nine highly involved, long-term female KBO spectators for a prior study on fandom formation were reanalyzed through systematic coding, employing the frameworks of bottom-up, hedonic, social, and eudaimonic well-being as structural guides. Results reveal a multidimensional paradigm of well-being generation: immediate hedonic well-being appears to be associated with dramatic game narratives and collective crowd dynamics, and relationships expand to foster social well-being through regional belonging and community solidarity. These dimensions shape the phenomenon of “fandom as an emotional sanctuary,” marked by collective identity integration and daily behavioral immersion. Despite threats to their well-being, fans employ active restoration strategies to re-engage. This pattern, observed within the bounded context of nine long-term KBO fans, points toward a possible pathway linking sustained fandom involvement to subjective well-being, characterized by emotional maturity and the assimilation of sports fandom into life’s meaning. These findings offer exploratory theoretical insights into the feminization of sports fandom and suggest that sports consumption may be relevant for modern psychological and social well-being.

## 1. Introduction

Launched in 1982, Korean professional baseball has grown into a representative national sport of South Korea over the past 40 years. In the league’s early days, a fandom linked to regional identity was formed based on a regional franchise system; however, as elements such as cheerleaders, cheerleading captains, and cheer songs took root during the league’s golden age in the 1990s, a prototype of the cheering culture that continues to this day was established (B. C. Kim, 2008). Subsequently, a culture of collective cheering spread throughout society, spurred by the 2002 Korea–Japan World Cup. Amid this trend, baseball stadiums have evolved from mere venues for watching games into complex spaces where cheering, communication, and leisure activities take place simultaneously. At present, professional baseball has established itself as a sociocultural leisure industry, transcending the realm of sports spectatorship (Jeong, 2025). This growth trend is evident in recent attendance statistics, with the overall average attendance in the 2024 season surpassing 10 million for the first time and with the 2025 season breaking the all-time record of 12.31 million (Jang & Lee, 2025).

Although various elements have likely contributed to this success, the influx of younger generations driven by the spread of new media and an increase in the female audience have been cited as key factors. A prime example is the KBO’s signing of a three-year broadcasting rights contract with an over-the-top platform, which allows for the free production and distribution of short video content, including game highlights, via YouTube and social media (Cho et al., 2026). In particular, the rapid expansion of the female fan base in their 20s and 30s over the past few years has been identified as one of the key driving forces behind this box office boom (Lee, 2025). In fact, the results of the 2024 professional baseball fan trend survey confirmed that the proportion of female spectators rose to 38%, women accounted for 54.4% of total ticket buyers, and their average spending per person exceeded that of male fans (Jeong, 2025). These fans are known to evolve into so-called “high-involvement fans” who go beyond simple ticket purchases to directly contribute to a club’s revenue generation through the consumption of expensive uniforms, various merchandise, and food and beverages; in this process, they form a unique fandom culture that is distinct from existing ones (Choi, 2025). Beyond their economic contribution, however, the psychological and social mechanisms through which this deepening engagement translates into personal well-being remain theoretically underexamined.

Baseball stadium culture, traditionally perceived as a male-dominated space, is being reorganized flexibly as a result of the influx of female fans. A notable first change is the spread of a consumption style where “live viewing” itself becomes the goal. Rather than focusing solely on the outcome of a game, female fans of winning and losing teams tend to consume the entire experience of enjoying food at stadiums and cheering with friends as a form of entertainment (Sveinson & Hoeber, 2016). This trend contributes to redefining baseball stadiums not only as spaces for watching games but also as complex spaces for cultural enjoyment. Another characteristic change is the pattern of passionate consumption and participation commonly observed in idol fandoms. Representative examples include immersing oneself in the individual narratives of players, singing cheer songs together, and injecting vitality into entire stadiums (Burroughs, 2020). In addition, actively expressing one’s fandom on social media using uniforms, dolls, and keyrings is a unique cultural characteristic of female fandoms; this pattern suggests that fans are forming a subculture through their own unique interpretations and modes of expression, going beyond simple sports “consumption” (Mercado & Bernthal, 2016). Given that female professional baseball fans are moving away from the position of passive spectators to actively enjoy and consume baseball culture in their own ways and are emerging as key actors driving the entire league, they are worth investigating through in-depth academic research.

However, existing studies on female fandom have generally focused on K-pop or idol fandom (Maros & Basek, 2022; Oh, 2015), while those exploring sports fandoms have focused on the experiences and perspectives of male fans (Mastromartino et al., 2025; Parry et al., 2014). Despite intermittent attempts to philosophically examine the feminization of sports fandom culture (Dodds et al., 2015) and study the process of overcoming the discriminatory gazes experienced by female professional baseball fans (Hoeber & Kerwin, 2013; Lee, 2025), existing studies have mainly focused on discrimination, stereotypes, and gender issues from a social constructivist perspective rather than closely examining the unique experiential characteristics of female fans. The process by which women become professional baseball fans and the process by which fandoms are formed are difficult to explain solely based on gender; rather, sociocultural backgrounds, such as external environmental factors surrounding the individual or internalized tendencies, can be regarded as having a more substantial influence (Hoeber & Kerwin, 2013). In this context, a recent qualitative study investigated the formation mechanism of female fandom using grounded theory, focusing on “how” female fans grow into high-involvement fans rather than on the motivational question of “why do female fans become high-involvement fans” (Yoon et al., 2026).

In particular, sport participation and spectating have been reported to have played an important role in maintaining mental health and alleviating emotional stress during the COVID-19 pandemic (Ma et al., 2025). Amid circumstances in which in-person leisure activities were broadly restricted, the emotional arousal and sense of community connection provided by sport spectating functioned as an important resource sustaining well-being in itself. As society moves into the post-pandemic era, amid deepening economic uncertainty, individualized work culture, and the restructuring of social networks, scholarly attention to well-being is being renewed, and sport is once again drawing attention as a key channel for promoting well-being (Delia et al., 2022).

Within this trend, high-involvement female fandom in professional baseball is a research subject that particularly merits attention. As discussed above, these fans are not passive spectators who merely consume game outcomes but active agents who are deeply engaged in the fandom experience through multiple layers—enjoying the live-viewing experience itself, immersing themselves in cheering activities, participating in fandom communities, and expressing their fan identity on social media. Given that such highly immersive experiences are likely to constitute a core everyday experiential resource that shapes well-being from a bottom-up perspective, there is a need to re-illuminate their fandom experiences through the new lens of well-being.

Therefore, the current study aims to reanalyze the fandom experiences of female professional baseball fans, drawing on raw data originally collected for a prior study on the fandom formation process (Yoon et al., 2026), through the theoretical lens of subjective well-being. Specifically, this study seeks to qualitatively explore the pathways through which the diverse experiential components constituting high-involvement fandom—emotional immersion, communal belonging, and identity integration—are connected to female spectators’ subjective well-being. In doing so, this study aims to elucidate the concrete mechanisms through which sport spectating experiences contribute to individuals’ quality of life and to provide theoretical and practical implications for enhancing the well-being of sport spectators in the post-COVID-19 era. It is important to clarify how the present study differs from and extends Yoon et al. (2026). Whereas the original study addressed the process-oriented question of how female fans progressively develop high-involvement fandom, the present study poses a conceptually distinct, outcome-oriented question: how such fandom, once established, shapes fans’ subjective well-being. Although the original interview protocol was not explicitly designed around well-being constructs, the semi-structured format elicited extensive first-person narratives concerning participants’ emotional states, relational experiences, identity, and life satisfaction associated with their fandom—material that is directly relevant to, and sufficiently rich for, a well-being-focused reanalysis. This reanalysis therefore does not simply reprocess the original findings but applies an independent analytical lens to previously uninterpreted dimensions of the same raw data, yielding a theoretical model and conclusions not reported in the original study. To this end, this study poses the following research questions:

RQ1: What role do the emotional and social experiences triggered by watching professional baseball play as resources for female fans’ hedonic and social well-being?

RQ2: Through what process does high-involvement fandom become integrated into female fans’ identity and daily life, thereby fostering their psychological well-being?

RQ3: How do threats to well-being arising from team slumps, player scandals, and internal conflicts within the fandom affect female fans, and through what strategies do they restore and sustain their well-being?

## 2. Literature Review

### 2.1. Sports Fandom

This literature review is organized to build cumulatively toward an integrated theoretical framework. Section 2.1 first establishes team identification as the psychological mechanism that binds an individual’s emotional state to their team’s fortunes. Section 2.2 then extends this foundation by reconceptualizing involvement not as a behavioral outcome but as the process through which this identification becomes embedded in daily life and self-concept. Section 2.3 subsequently synthesizes these two literatures, together with the well-being literature, to derive the theoretical model that structures the analysis in Section 4.

Sports fandom is a psychosocial phenomenon that goes beyond the mere act of watching games and involves the consumption of sports as a part of one’s self-identity and emotional attachment to specific teams or players. Whereas spectators are consumers interested in game results or acquiring information, fans exhibit repetitive consumption behaviors based on sustained interest and emotional immersion in the subject (Parry et al., 2014). This distinction aligns with recent theoretical trends that view fandom as a continuum rather than a single category, focusing on the “quality of engagement” rather than the “frequency” of viewing (Melnick & Wann, 2011). The concept of team identification has long been used as a core explanatory framework in sports fandom research (Norris et al., 2015). Specifically, team identification refers to the degree to which an individual internalizes a specific team as part of their social identity; the higher the level of identification, the stronger the tendency to link the team’s success or failure to one’s psychological well-being. This concept of identification has served as the theoretical foundation for the developmental perspective, which understands fandom as a stepwise developmental process of “interest → attachment → involvement → loyalty” (Kung & Klein, 2025). Because a strong sense of team identification ties the outcome of a game directly to one’s own emotional state, this body of work provides an important theoretical starting point for understanding how the process of fandom development is inherently intertwined with an individual’s psychological experience. As elaborated in Section 2.3, this identification-based coupling of team outcomes with personal affect is theorized here specifically as a pathway to hedonic well-being (through the emotional highs and lows of game outcomes) and psychological well-being (through the gradual incorporation of the team into one’s self-concept), rather than as a generalized or undifferentiated affective response.

### 2.2. Involvement Theory

Involvement is defined as the perceived degree of importance and relevance that a specific object or activity holds for an individual. In consumer behavior research, involvement has been widely used as a core concept explaining attitude formation and purchasing decision-making. In the case of female professional baseball fans, they directly contribute to club revenue generation by going beyond simple ticket purchases and consuming expensive uniforms, various merchandise, and food and beverages (Osborne & Coombs, 2013). In fact, the average per capita spending of female spectators has been confirmed to exceed that of male fans (Gemar & Pope, 2022). This expansion of consumption behavior suggests that the rise in involvement levels is not merely an increase in spending but is intertwined with a psychological process in which fan identity is integrated as part of the self-concept. From the perspective of social identity theory, deepening involvement can be understood as a process in which an individual accepts a sense of belonging to a specific group (fandom) as part of their self-definition. Given that involvement research has traditionally centered on behavioral and attitudinal outcomes such as spending and loyalty, less attention has been paid to how this psychological integration of fan identity into the self-concept translates into affective and evaluative outcomes for the individual. This is precisely the gap addressed in Section 2.3: involvement is reconceptualized here not merely as a behavioral or attitudinal outcome but as a mechanism through which fan identity becomes integrated into the self, thereby contributing to both social well-being (via group belonging) and psychological well-being (via identity integration).

### 2.3. Sports Fandom, Involvement, and Subjective Well-Being

Before proceeding, it is necessary to clarify the conceptual boundaries among the well-being constructs examined in this study, as these terms are often used inconsistently across the literature. Subjective well-being is the overarching, self-reported evaluation of one’s life, encompassing both affective and cognitive components (Diener, 1984). Within this umbrella construct, hedonic well-being refers to the presence of positive affect and pleasure, typically arising from immediate, sensory, or emotionally arousing experiences. Eudaimonic well-being, in contrast, denotes a deeper and more enduring sense of meaning, purpose, and self-realization that develops through sustained engagement rather than momentary pleasure. Social well-being captures the relational dimension of well-being, namely, the sense of belonging, integration, and solidarity that an individual derives from group membership and interpersonal ties. Psychological well-being refers to the integration of a valued social identity into one’s broader self-concept, contributing to a stable and coherent sense of self. In the present study, these four constructs, hedonic, social, psychological, and eudaimonic well-being, are treated as interrelated but conceptually distinct components that jointly constitute the broader construct of subjective well-being, and each term is used consistently throughout the Results and Discussion to label the specific well-being outcome under discussion.

The preceding discussions of fandom and involvement converge on a common implication: as fans become more deeply identified with and involved in a team, the boundary between the fan’s self-concept and the fan object becomes increasingly blurred, such that the team’s fortunes come to bear directly on the individual’s emotional and psychological state. This convergence provides the theoretical rationale for extending fandom and involvement research into the domain of subjective well-being. Discussions of well-being are broadly divided into two approaches: the top-down approach, which regards well-being as determined by an individual’s stable internal disposition or external factors such as material wealth, and the bottom-up approach, which understands well-being as the cumulative sum of an individual’s evaluations of the diverse life experiences accumulated in daily life (Diener, 1984). Sport-related studies have primarily adopted the latter perspective, examining how high-quality sport participation and spectating experiences contribute to the formation of subjective well-being among participants and spectators (J. Kim & James, 2019; Silva et al., 2020; Kumai et al., 2025).

Within this framework, the present study positions team identification and fan involvement not merely as antecedents of loyalty or consumption behavior, but as psychological mechanisms through which fandom experiences are converted into resources for hedonic well-being (immediate positive affect derived from game-watching experiences), social well-being (a sense of belonging and solidarity formed through fan communities), and psychological well-being (self-identity integration through team identification and sustained involvement). This integrated perspective addresses a gap in the existing literature, which has examined fandom formation, involvement, and well-being largely as separate lines of inquiry, and provides the theoretical foundation for the present study’s reanalysis of high-involvement female fandom through the lens of subjective well-being.

## 3. Research Methods

### 3.1. Operational Definition

In defining the female fans participating in this study, the classification criteria of Shank and Beasley (1998) were applied to establish an operational definition of “highly involved fans.” Specifically, a “highly involved female fan of professional baseball” was defined in this study as follows: first, a person who is aware of the previous season’s champion of the professional baseball league in which they are interested; second, a person who knows the names of all players belonging to the team they support; and third, a person who shows a high level of involvement to the extent that they own the uniform of the team they support.

### 3.2. Research Participants

The study participants comprised nine devoted female fans of professional baseball who fit the operational definition presented earlier, had consistently watched professional baseball in person for at least 5 to 10 years, and had never changed the team they support. They were selected using the purposeful sampling method, which was intended to enhance the authenticity of the data and secure in-depth information in specific contexts by limiting the scope of participation to specific conditions (Eyler et al., 1998). The participants’ specific characteristics are listed in Table 1. This secondary analysis was conducted under a separate ethical approval (IRB-HYU-2026-177) obtained specifically for the reanalysis of the raw interview data. Informed consent for the original data collection had been obtained from all research participants at the time of the original study (Yoon et al., 2026).

### 3.3. Data Collection and Analysis

Raw data were collected through in-depth interviews conducted with nine research participants over a period of approximately three months from 15 September to 20 November 2025. These raw data were originally collected for a prior study on the fandom formation process (Yoon et al., 2026) and were reanalyzed in the present study through the theoretical lens of subjective well-being. Data collection was conducted through the recording of the overall atmosphere of the in-depth interview sites, observation records in each situation, the flow of the overall narrative regarding the process of fandom formation, and repeated in-depth interviews with the participants. Various episodes occurring during the fandom formation process were recorded and stored in observation logs.

To prioritize participants’ psychological comfort and minimize evaluative apprehension, participants were given full autonomy to select a location in which they felt most able to share their experiences candidly, such as a private university seminar room, a quiet meeting room, or a quiet café; where geographic or scheduling constraints arose, interviews were conducted via a secure real-time video conferencing platform (Zoom), such that both in-person and remote formats were used as appropriate. Each participant took part in one to two individual, semi-structured in-depth interviews, with each session lasting approximately 60 to 90 min (total interview time per participant ranging from 70 to 100 min). Interviews followed a prepared standard protocol but incorporated flexible, structured probing questions tailored to each participant’s responses in order to elicit rich, contextualized narratives. Prior to data collection, all participants received a detailed explanation of the study’s purpose, the anonymization and confidentiality of their data, and their right to withdraw, and written informed consent was obtained. With participants’ prior consent, interviews were recorded in their entirety using a high-quality digital audio recorder, and the resulting audio files were transcribed verbatim within 24 h. To further establish the trustworthiness of the data and the validity of the interpretation, member checking was conducted with participants as needed after transcripts were completed.

Although the original semi-structured interview protocol was designed to elicit narratives of fandom formation rather than well-being per se, its six thematic sections in fact correspond closely to the well-being dimensions examined in this study: hedonic arousal and catharsis experienced at the stadium, the extent to which fandom is woven into daily routines, relational bonds and identity formed through fandom, threats to well-being such as team slumps, player scandals, and gendered stigma together with participants’ coping responses, and the ultimate meaning of fandom in participants’ lives. Participants were explicitly asked whether baseball functioned as a mere hobby or as an “emotional sanctuary” protecting their soul, a phrase that directly informed the central phenomenon identified in Section 4.3 of the present study. This structural correspondence, rather than a post hoc reading of the transcripts alone, establishes the adequacy of the original data for addressing the present study’s research questions.

Given the theory-driven nature of this reanalysis, in which raw data collected for a different research purpose were reexamined using subjective well-being as an explicit analytical lens, the present study is best characterized as a secondary qualitative analysis informed by grounded-theory-derived coding procedures, rather than a grounded theory study in the classical inductive sense. This distinction is made explicit here because grounded theory, in its traditional form, generates theoretical categories inductively from data collected specifically for that purpose; the present study, by contrast, applies grounded-theory-style open, axial, and constant-comparative coding techniques to systematically organize the data while remaining guided by an a priori theoretical framework. This hybrid approach was adopted because it allowed the systematic, bottom-up organization of participants’ own words into categories, while still permitting the resulting categories to be interpreted through, and connected to, an explicit theoretical structure.

A new analysis procedure was also performed on the in-depth interview transcripts and raw observation log data. Specifically, unlike the existing coding of Yoon et al. (2026), which aimed at identifying the ‘formation process’ of fandoms, this study conducted re-open coding on the same raw data using subjective well-being theory as the analytical criterion. In this process, the research team re-extracted statements directly related to well-being from the participants’ remarks, such as emotional arousal, social bonding, sense of belonging, identity integration, and daily satisfaction; subsequently, these statements were re-categorized based on the bottom-up concept of well-being and the theoretical distinction between hedonic and eudaimonic well-being (Diener, 1984). The re-categorized concepts were subsequently integrated into higher categories through an axial coding stage, and the relationships between the categories were refined using a constant comparative method.

All coding was conducted collaboratively and manually by the three co-authors, who met on multiple occasions to jointly review, code, and discuss the Korean-language transcripts, rather than coding independently and later reconciling discrepancies. Coding was performed manually rather than through qualitative analysis software in order to preserve the nuance, idiomatic expression, and cultural specificity of the original Korean interview data, which would otherwise risk being lost or distorted through translation. Analytical agreement was reached through iterative group discussion until consensus was achieved on each category and its defining properties, a process that also served as a form of ongoing investigator triangulation. Data collection and analysis continued until no substantially new well-being-related categories or properties emerged from successive transcripts, at which point the nine cases were judged to have provided sufficient conceptual richness for the categories identified in Section 4.

## 4. Results and Discussion

**Figure 1:**
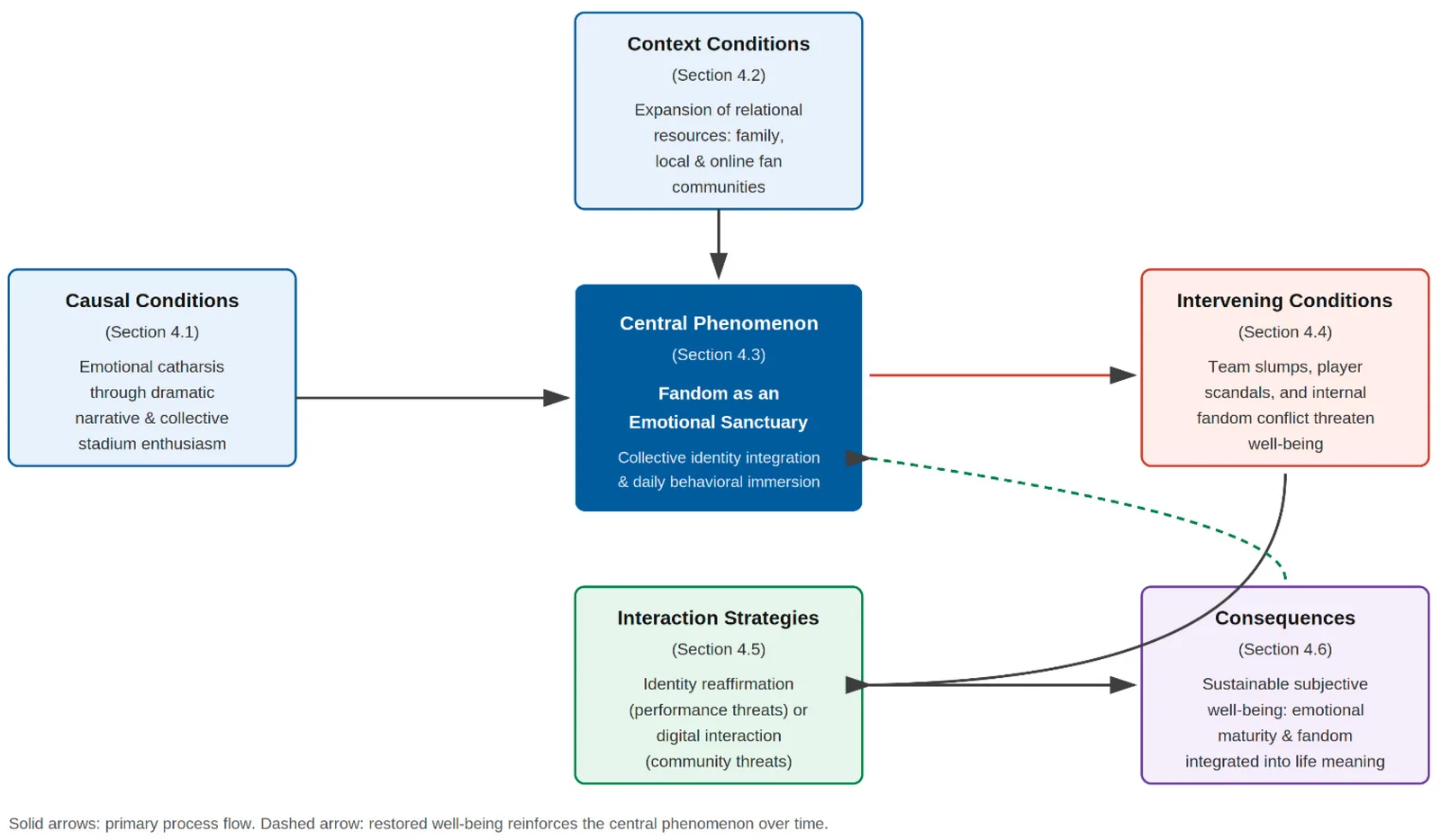
The well-being formation process of highly involved female KBO fans, illustrating the relationships among the six categories (Section 4.1, Section 4.2, Section 4.3, Section 4.4, Section 4.5 and Section 4.6) derived from the analysis.

Figure 1 provides a visual overview of the well-being formation process derived from the analysis, illustrating how the six categories identified below are related to one another; each category is discussed in detail in the corresponding subsection.

### 4.1. Causal Conditions: Sources of Hedonic Well-Being

Regarding the background of the source of hedonic well-being derived as a causal condition, this study confirms that the cause of the central phenomenon unfolds in two aspects: “emotional catharsis through dramatic narrative” and “immersion overwhelmed by the collective enthusiasm of the scene.”

*Going to the stadium in person makes me incredibly excited. Before even entering, I feel overwhelmed by the sound of the cheering songs, the energy inside, and the vast space. I love the feeling of my heart pounding just from hearing the cheers and the atmosphere where you sing, drink beer, and become friends with strangers.*

(Research Participant I)

*A walk-off hit with two outs and the bases loaded in the 9th inning! It’s just a dopamine-pumping moment.*

(Research Participant A)

*I love seeing everyone cheering so energetically together. With basketball, you have to focus on the game rather than the cheering, but baseball has an atmosphere where everyone enjoys themselves. For our Lotte team, using orange bags to sing “Busan Seagull” really relieves stress, and going to the stadium is just so exciting. And above all, it feels like we’re breathing together with the players.*

(Research Participant B)

*I think I became a fan ever since watching my first game live. At first, my friends were skeptical, asking, “Do you really know baseball?”, but these days, they’ve given up on that idea. I don’t even make plans on days when I have a game.*

(Research Participant H)

The in-depth interviews with the study participants revealed that emotional arousal triggered by watching games served as an experiential resource that repeatedly provided the participants with intense and immediate positive emotions, functioning as an important resource for hedonic well-being. These results partially align with those of Cronin and Cocker (2019), who found that emotional arousal and bonding between the supported team and the fans are maximized through group cheering, singing, and gestures before and after the game. Furthermore, this study is supported by the fact that it directly aligns with the dimension of positive affect among the components of subjective well-being proposed by Diener (1984).

Particularly noteworthy is that the emotional arousal mentioned by the participants did not stem solely from the outcome of victory but was distributed throughout the entire course of the game, including through fan songs, shouts, and spontaneous kinship among strangers. This result aligns with the discussion by Han et al. (2016), who viewed high-quality sports viewing experiences as contributing to the formation of subjective well-being in participants and spectators, and with the study of Kumai et al. (2025), who identified the pathways through which multidimensional experiences provided by sports consumption activities contribute to well-being. In other words, the results of the current study also support the idea that subjective well-being does not stem from a single outcome-oriented event but rather manifests through the accumulation of an individual’s cognitive perceptions of a high-quality sports environment, as pointed out by Inoue et al. (2020). The immediate pleasure that female fans experience by watching games clearly begins to function not as a random, one-time experience but as a continuous emotional resource that is repeatedly recalled and reinforced.

Notably, several of these hedonic responses are closely tied to cheering practices distinctive to Korean baseball culture, such as coordinated chants (ttechang), team-specific cheer songs, and cheerleader-led stadium choreography, which differ markedly from the more individualized spectatorship patterns typical of many Western sports contexts. This suggests that the specific form through which hedonic well-being is generated in this study is not merely a generic byproduct of sport spectating but is substantially shaped by a culturally specific mode of collective participation, a point that warrants caution when generalizing the present findings to fan cultures organized around different cheering conventions.

### 4.2. Context Conditions: Expansion of Relationships Leading to Social Well-Being

Along with causal conditions, the situational context that directly influences the central phenomenon falls under the higher category of the expansion of relationships that leads to social well-being. This category consists of three relational resources: “formation of initial relationship-based bonds,” “sense of belonging through local communities,” and “sense of solidarity through on-site and online communities.” These relational resources serve as social turning points, transforming research participants from individual spectators into fans belonging to a community through a single experience.

*At the age of seven, watching a game live at Daegu Stadium with my father was the starting point of my first experience as a baseball fan. Since my hometown is Daegu, I naturally became a Samsung fan, and as I frequently attended games with friends during high school, you could say I became a total fan.*

(Research Participant E)

*I remember cheering outside the stadium with some middle-aged men while eating chicken because the tickets were sold out. I even used the cheer song as my ringtone. Wouldn’t you say that makes you a true fan?*

(Research Participant H)

The results of such relational stimulation can be explained as an expansion of social bonding, which is partially consistent with the study of Russell (2012), who stated that local identity is formed through connections with the local community and becomes the basis of fandom through the process of attachment to the local sports team. The results are also supported by the findings of Samra and Wos (2014), who showed that the regional alignment between the sports team and the consumer has a significant influence on becoming a fan, and those of Ko et al. (2022), who showed that children in areas with very strong local fandom are complexly influenced by family fandom.

Interpreted through the well-being framework established in Section 2.3, these relational resources are best understood as the mechanism through which social well-being, specifically the sense of belonging and solidarity derived from group membership, is generated for female KBO fans. Rather than functioning as a discrete or one-time event, family- and community-based relational bonds appear to operate cumulatively: an initial relationship-based entry point (e.g., attending games with a parent) becomes reinforced through repeated participation in local and online fan communities, gradually consolidating into a stable relational foundation upon which the more identity-integrated forms of well-being discussed in Section 4.3 are built.

### 4.3. Central Phenomenon: Fandom as an Emotional Sanctuary

Fandom as an emotional sanctuary, the central phenomenon of this study, serves as a point where all emotions and experiences of well-being felt by female fans are concentrated. Psychological well-being is triggered, and a foundation for maintaining long-term well-being is formed through the complex interaction of the aforementioned causal conditions and situational contexts regarding the two aspects of “integration into collective identity” and “continuous immersion as a rhythm of life.”

*The moment I join the cheering squad, I cease to be an individual and become part of the collective identity of a “Lions fan.” We cheer until our voices burst and high-five the person next to us; if I go alone, I can’t last until the 9th inning, but if we go together, we can make it even to the 12th.*

(Research Participant F)

*Our team is always like a drama, isn’t it? We share a narrative of “we eventually make it happen.” It is the shared values of comforting and uniting with each other—thinking that even if we don’t win, there is value in being together like this—that sustain the fandom.*

(Research Participant I)

*I communicate via Instagram or Open Chat, and whenever I go to a game in person, I always make sure to upload short videos like a vlog. I do it with the mindset of recording history as a fan.*

(Research Participant G)

According to the results of the in-depth interviews with the study participants, the central phenomenon, which can be considered the core of this research, was the formation of a fandom as an emotional sanctuary through a process of integration into a collective identity and becoming established as a rhythm of life. Fans define themselves by the collective identity of being “fans of the team” rather than by their individual names, forming emotional bonds that maximize a sense of belonging within a well-being community (Chun & Sagas, 2022; Fink et al., 2009).

These results align directly with the bottom-up perspective (Diener, 1984) in theoretical discussions of well-being, which is understood as the cumulative evaluation of an individual’s life. In particular, the identity integration and daily immersion demonstrated by the participants strongly embody the characteristics of eudaimonic well-being, which goes beyond mere hedonic pleasure and provides lasting meaning and purpose to an individual’s life. The results are also aligned with the study of Silva et al. (2020), which focused on the process by which sports participation and viewing contribute to the subjective well-being of participants and spectators, and with the findings of Kumai et al. (2025), who developed a multidimensional well-being scale targeting professional baseball fandom. In other words, the fandom as an emotional sanctuary revealed in this study functions as a sustainable psychological well-being resource that shapes self-identity and life rhythms, going beyond being merely a source of temporary pleasure for participants; through this process, it becomes a key driving force in the formation of sustainable psychological well-being.

### 4.4. Intervening Conditions: Conflict Factors Threatening Well-Being

In the process by which female fans form psychological well-being, the mediating conditions were identified as “emotional burnout due to poor team performance,” “a sense of betrayal and identity confusion caused by player scandals,” and “damage to the sense of belonging due to internal divisions within the fandom.”

*We won back-to-back games frequently early in the season. However, we failed to advance to the postseason again. Do you know what other teams call us? They call us a team that has good momentum early in the season (spring) but sees its performance plummet once summer passes. It is inevitable to feel disappointed when the team is struggling.*

(Research Participant I)

*It is hardest to see our fans fighting among themselves. If you are a true fan, I wish you wouldn’t use abusive language. The more things are like this, the more we need to stick together. I wish the fake fans would leave now. We don’t need fake fans who have no identity for our team.*

(Research Participant A)

The research participants clearly experienced emotional exhaustion as they faced mediating conditions, such as their supported teams’ poor performance or the splitting of the fandom, while their psychological well-being was gradually threatened in the fandom as an emotional sanctuary, which was the central phenomenon mentioned earlier. This result is supported by the findings of Onwe (2016), who stated that positive emotions such as joy and pride are strongly induced when the supported team wins while negative emotions such as frustration and anger are prominent when they lose.

### 4.5. Interaction Strategies: Restoring Well-Being

A closer examination of the data indicates that the specific threat encountered shaped the specific restoration strategy adopted. Threats stemming from poor team performance, which participants experienced as a diffuse form of emotional fatigue rather than a rupture in belonging, were primarily met with identity reaffirmation; participants doubled down on their collective fan identity rather than distancing themselves from it. In contrast, threats stemming from internal fandom conflict, which more directly damaged participants’ sense of belonging to the fan community itself, were more often addressed through digital interaction; that is, participants sought to rebuild a sense of connection through community engagement on social media and messaging platforms. This differentiated pattern suggests that restoration strategies are not applied uniformly but are calibrated to the specific well-being dimension under threat.

The participants shared how they re-immersed themselves as fans by restoring well-being as an interaction strategy in the process of responding to negative realities, such as poor performance, scandals, and internal divisions, which acted as obstacles in the central phenomenon of fandom as an emotional sanctuary. Specifically, “strengthening community solidarity through identity reaffirmation” and “emotional recharging through digital interaction” were analyzed as interaction strategies.

*Our team is always like a drama, isn’t it? Our shared values of comforting and sticking together, saying that even if we don’t win, there is value in being together like this, are what sustain our fandom.*

(Research Participant I)

*I was incredibly stressed because I couldn’t attend games in person after being transferred to Jeju. Now that I have more free time, I try to go to as many games as possible. Last year, I even took annual leave to make sure I followed the team all the way to the away game in Gwangju. It really isn’t easy to watch a game on the mainland from Jeju, so isn’t that amazing?*

(Research Participant F)

The participants also gradually revealed themselves as emotionally mature fans as they went through a series of processes to restore their well-being within the fandom, which serves as an emotional sanctuary, despite their supported teams’ slump, scandals involving some players, and internal divisions. Female professional baseball fans play a significant role in restoring well-being by transforming from mere spectators into active fans who engage in cheering, community activities, and content production (Yoshida et al., 2014). In particular, the case of a participant who overcomes realistic constraints, such as physical distance (being assigned to Jeju Island), by engaging in more active fan activities (taking annual leave to attend away games) demonstrates that high-involvement fandom has established itself as a life priority in that fans voluntarily invest time and resources to protect, going beyond enjoying a simple hobby.

### 4.6. Consequences: Sustainable Subjective Well-Being

The individual outcomes observed in the series of processes regarding the formation of well-being within the female professional baseball fandom were found to be ultimately integrated into sustainable subjective well-being. This finding was confirmed as a consequent meaning where well-being takes root in one’s entire life, characterized by “stable immersion through emotional maturity” and “well-being integrated into the meaning of life.”

*In the past, I used to just cheer, but now I accept it calmly even if we lose. As the season approaches, I find myself making plans to attend games in person with a relaxed attitude.*

(Research Participant I)

*If we had lost the Korean Series to Hanwha (though that’s unlikely to happen), I probably would have just stayed home and drunk from the shock. That’s how much the team’s results affect my emotional state. Once a fan, always a fan.*

(Research Participant G)

*I really teared up when we won last year and sang the cheering song. I wore the glossy jacket my father bought for me back in college and cried my eyes out. A few years ago, I had lost my father, who was also a fan. At that moment, I felt like I was with my dad, so I was both very happy and sad.*

(Research Participant H)

*Being a fan is no longer a hobby; it has become my very life. To the point where I even save proof shots in a dedicated “Lotte Collection” folder on social media.*

(Research Participant B)

A close examination of the well-being formation process of the female professional baseball fandom revealed that repeated experiences of threats actually lead to the development of psychological adaptation mechanisms for maintaining well-being. This result aligns with the discussion of J. Kim and James (2019), who viewed sports participation as contributing to participants’ short- and long-term subjective well-being and the fulfillment of psychological needs. In particular, the emotional adaptation patterns of the participants who maintain their fandom even amid repeated frustrations empirically demonstrate that well-being is not a fixed psychological characteristic but a process that develops through the accumulation of experiences.

A particularly noteworthy finding emerged from the statement of a participant who shed tears while recalling her lost father at the moment of victory. This experience demonstrates that watching sports can function as a channel to restore emotional connections with lost family members and reconstruct the meaning of life, going beyond mere entertainment consumption. Such an experience can be understood as eudaimonic well-being—that is, an experience that imbues deep meaning into an individual’s life—transcending the dimension of hedonic well-being. This result further suggests that the influence of watching sports on an individual’s emotional life extends beyond superficial pleasure. It also connects to the discussion by J. Kim et al. (2017), who viewed sports consumption experiences as contributing to the formation of participants’ life meaning and identity.

In summary, the process of well-being formation among the highly engaged female professional baseball fans revealed in this study can be understood as a dynamic cyclical structure in which positive resources along three axes, namely, hedonic well-being (immediate emotional resources), social well-being (expansion of relationships and belonging), and psychological well-being (identity integration and self-actualization), are shaken by threat factors such as poor performance, scandals, and internal conflicts but are reintegrated into sustainable well-being through recovery strategies such as identity reaffirmation and digital interaction. These results provide concrete and in-depth support for the perspective of previous studies (Kumai et al., 2025; Silva et al., 2020) that sports participation and viewing contribute to the subjective well-being of participants and spectators, specifically targeting highly engaged female fans. Furthermore, on the basis of the discussions during the pandemic that sports spectating played an important role in maintaining mental health and alleviating emotional stress (Ma et al., 2025), the patterns of emotional recovery and reintegration through fandom that were demonstrated by the participants of the current study can be interpreted as empirical evidence supporting the potential of sports spectating as a channel for promoting well-being in the post-COVID era.

Taken together, these six categories illustrate that hedonic, social, psychological, and eudaimonic well-being, as conceptually distinguished in Section 2.3, are not independent outcomes but interlocking components of a single developmental trajectory: immediate hedonic experiences accumulate into relational and psychological resources, which are periodically destabilized by identifiable threats and subsequently restored through targeted strategies, ultimately consolidating into the more enduring, meaning-oriented form of well-being captured by the eudaimonic construct. This trajectory should be interpreted as a pattern observed within the bounded context of nine long-term, high-involvement KBO fans embedded in a culturally distinctive cheering environment, rather than as a universal or linear causal sequence generalizable beyond this context.

## 5. Conclusions

The purpose of this study was to analyze, using a secondary qualitative analysis informed by grounded-theory coding procedures, the pathways through which the high-involvement fandom experiences of female professional baseball fans contribute to their subjective well-being as the fandom becomes firmly established and to derive a theoretical explanatory framework. Based on the objectives of this study and the analyzed results, the following conclusions were drawn.

First, regarding the causal conditions, the “source of hedonic well-being” was identified as having subcategories of “emotional catharsis through dramatic narratives” and “immersion overwhelmed by the collective fervor of the venue.” The emotional arousal triggered by watching a game act was found to serve as an experiential resource that repeatedly provides the participants with intense and immediate positive emotions. Furthermore, this immediate pleasure begins to function not as a random, one-time experience but as a continuous emotional resource that is repeatedly recalled and reinforced.

Second, regarding the situational context, the “expansion of relationships leading to social well-being” was identified as having subcategories of “formation of initial relationship-based bonds,” “sense of belonging through local communities,” and “sense of solidarity through on-site and online communities.” The result indicates that relational resources transform individual spectators into fans belonging to a community. The result also reveals that the initial bonds formed through family and acquaintances expand into a sense of solidarity through on-site and online communities, and this social well-being holds significance as a resource that complements relational deficiencies in the post-COVID era.

Third, regarding the central phenomenon of “fandom as an emotional sanctuary,” “integration into a collective identity” and “continuous immersion as a rhythm of life” were derived as subcategories. Causal conditions and situational contexts were found to interact in a complex manner to trigger psychological well-being and form the foundation for maintaining long-term well-being. Fans define themselves through a collective identity that transcends individual identity, and through this process, they serve as the core driving force for the formation of continuous psychological well-being.

Fourth, regarding the mediating conditions, the “conflict factors threatening well-being” were identified as the following subcategories: “emotional exhaustion due to poor team performance,” “sense of betrayal and identity confusion due to player scandals,” and “damage to the sense of belonging due to internal rifts within the fandom.” This result confirms that individual fans’ well-being is not entirely within their own control and can be significantly influenced by external events concerning the object of their support. In particular, this study found that internal rifts within the fandom act as a more fundamental threat that undermines the sense of belonging itself compared with threats caused by external factors.

Fifth, regarding the interaction strategy “recovering well-being,” “strengthening community solidarity through identity reaffirmation” and “emotional recharging through digital interaction” were identified as subcategories. The participants demonstrated the qualities of agents who actively recover their well-being through proactive strategies rather than passively being exposed to negative events that threaten their well-being. As discussed in Section 4.5, the specific strategy adopted was not uniform but corresponded to the specific well-being dimension under threat, with performance-related threats prompting identity reaffirmation and community-related threats prompting digital interaction.

Sixth, the results confirmed that “sustainable subjective well-being” refers to “stable immersion through emotional maturity” and “well-being integrated into the meaning of life,” implying that repeated experiences of threat actually lead to the development of psychological adaptation mechanisms to maintain well-being. Particularly, the experience of restoring emotional connections with lost family members demonstrates that watching sports can function as a pathway to eudaimonic well-being by transcending the hedonic dimension.

This study suggests the need to understand the high-involvement female professional baseball fandom not merely as a leisure consumption activity but also as a dynamic pathway for well-being formation, where immediate emotional resources (hedonic well-being), relational resources (social well-being), and identity resources (psychological well-being) are integrated into one’s entire life through a cyclical process of threat and recovery. Specifically, given the finding in Section 4.2 that relational entry points such as attending games with family members served as a foundation for social well-being, clubs could consider organizing family-oriented events that provide similar structured opportunities for newer fans. Given the finding in Section 4.4 that internal fandom conflict was identified by participants as a more fundamental threat to belonging than external, performance-related setbacks, this suggests that attention to the health of fan communities themselves, and not only to team performance, may be a relevant consideration for clubs. Given the finding in Section 4.5 that participants relied on digital interaction to restore a sense of connection during periods of strain, this points to the potential value of the informal digital spaces that fans already use for this purpose.

They must also establish an environment that supports their female fans’ emotional experiences, communication channels to mediate internal conflicts within the fandom, and fan communication strategies for crisis situations.

This study is subject to several limitations. First, because the data analyzed were originally collected for a study with a different research focus (fandom formation, Yoon et al., 2026), the interview protocol was not specifically designed to elicit well-being-related content; although the structural correspondence between the protocol and the well-being dimensions examined here, described in Section 3.3, supports the adequacy of the data, some dimensions of well-being that a purpose-built interview protocol might have captured cannot be ruled out as underrepresented in the present data. Second, the sample consisted exclusively of nine long-term, high-involvement female fans who had never changed their supported team; this homogeneity, while appropriate for the study’s aim of understanding well-being among established high-involvement fans, limits the transferability of the findings to low-involvement, casual, or newly converted female fans, whose well-being pathways may differ substantially. Third, participants’ accounts may have been shaped by their team’s performance and their personal circumstances at the specific point in time when the original interviews were conducted (September to November 2025); fans interviewed during a different phase of the season, or during a different period of their personal lives, might report a different balance of hedonic, social, and threat-related experiences.

Finally, this study suggests areas for future research. First, while this study explored the qualitative path between high-involvement fandom and well-being through secondary qualitative analysis informed by grounded-theory procedures, analyzing quantitative data using well-being scales on a broader range of female fans through quantitative research would aid in statistically verifying the theoretical path presented in this study. Second, although this study explored the well-being experiences of highly involved female fans, future research is needed to more precisely elucidate the relationship between involvement and well-being through comparative studies of well-being across various levels of involvement, such as low-involvement and potential fans. Third, as this study focused on professional baseball as a single sport, the universality and specificity of the well-being formation paths derived from this study must be verified across different sports through comparative studies targeting female fandoms in other sports such as soccer and basketball.

## Figures and Tables

**Table 1:** Research participants.

No.	Name	Occupation	Fan Career	Viewing Frequency (Year)	Supported Team
A	Park, S	Office worker	16 years	10 times+	Kia
B	Shin, H	Office worker	6 years	5 times+	Lotte
C	Kim, S	Full-time housewife	10 years	10 times+	Hanwha
D	Yoo, S	Working mom	15 years	10 times+	SSG
E	Kim, S	Office worker	15 years	10 times+	Samsung
F	Kim, Y	Office worker	25 years	20 times+	Samsung
G	Kwon, H	Beauty shop owner	10 years	10 times+	LG
H	Kim, J	Graduate student	15 years	15 times+	LG
I	Kim, S	University student	11 years	20 times+	Lotte

## Data Availability

The data presented in this study were originally collected for Yoon et al. (2026) and are not publicly available due to participant confidentiality agreements. Requests to access the datasets should be directed to the corresponding authors.
